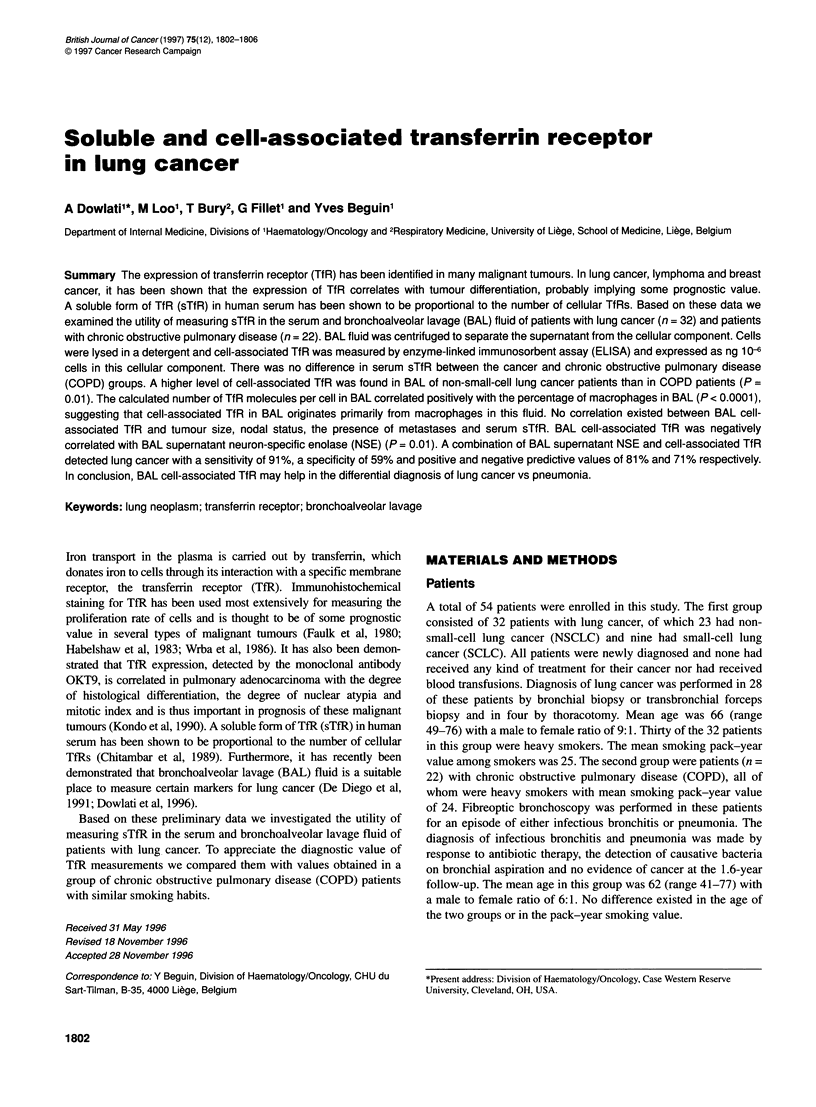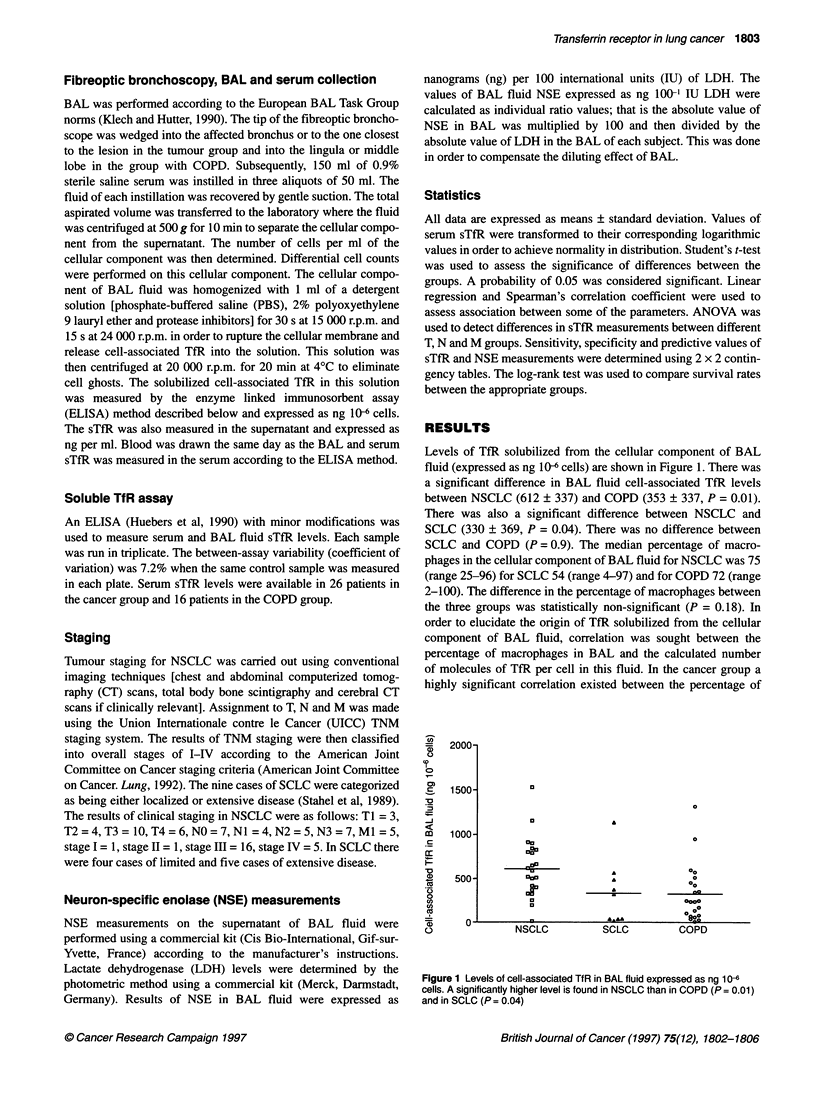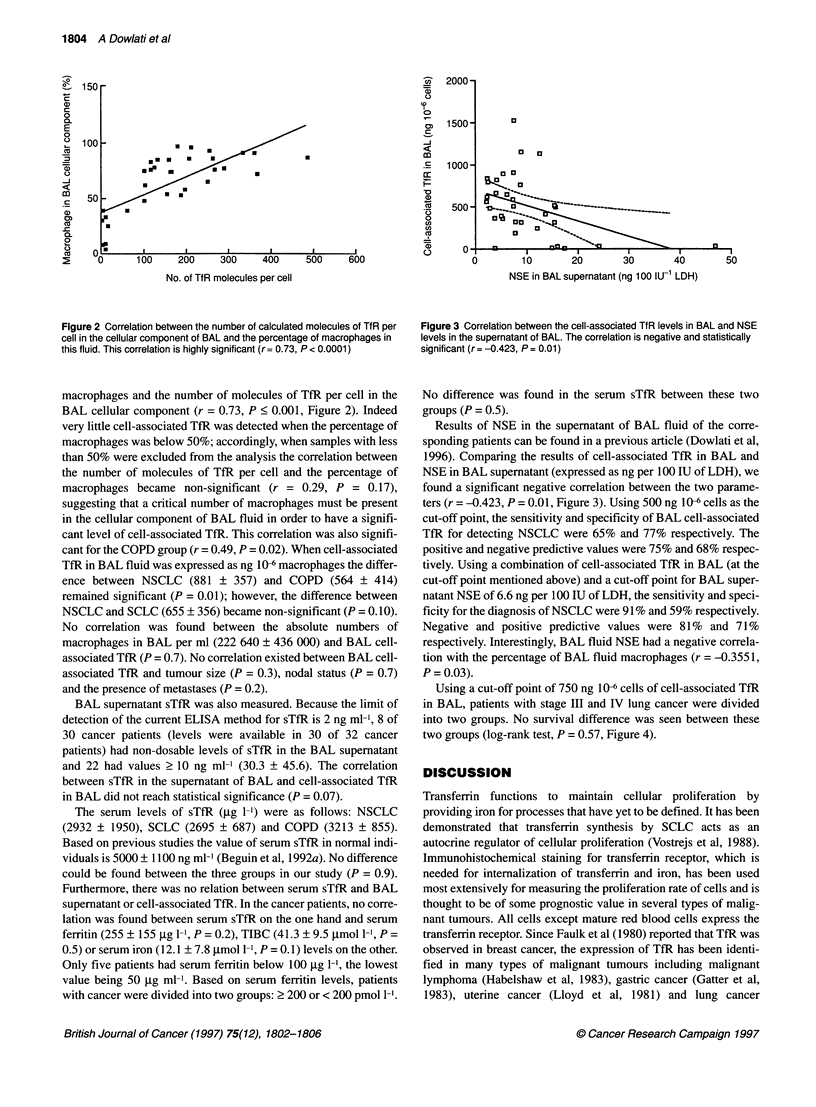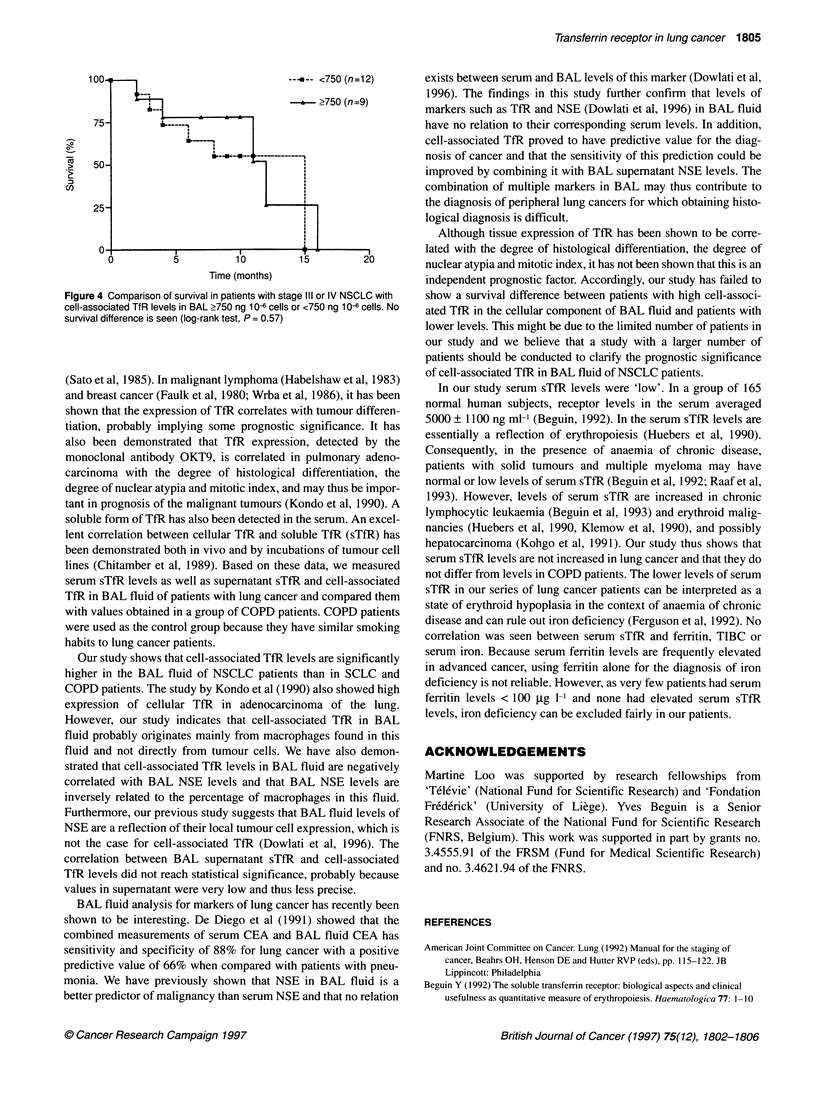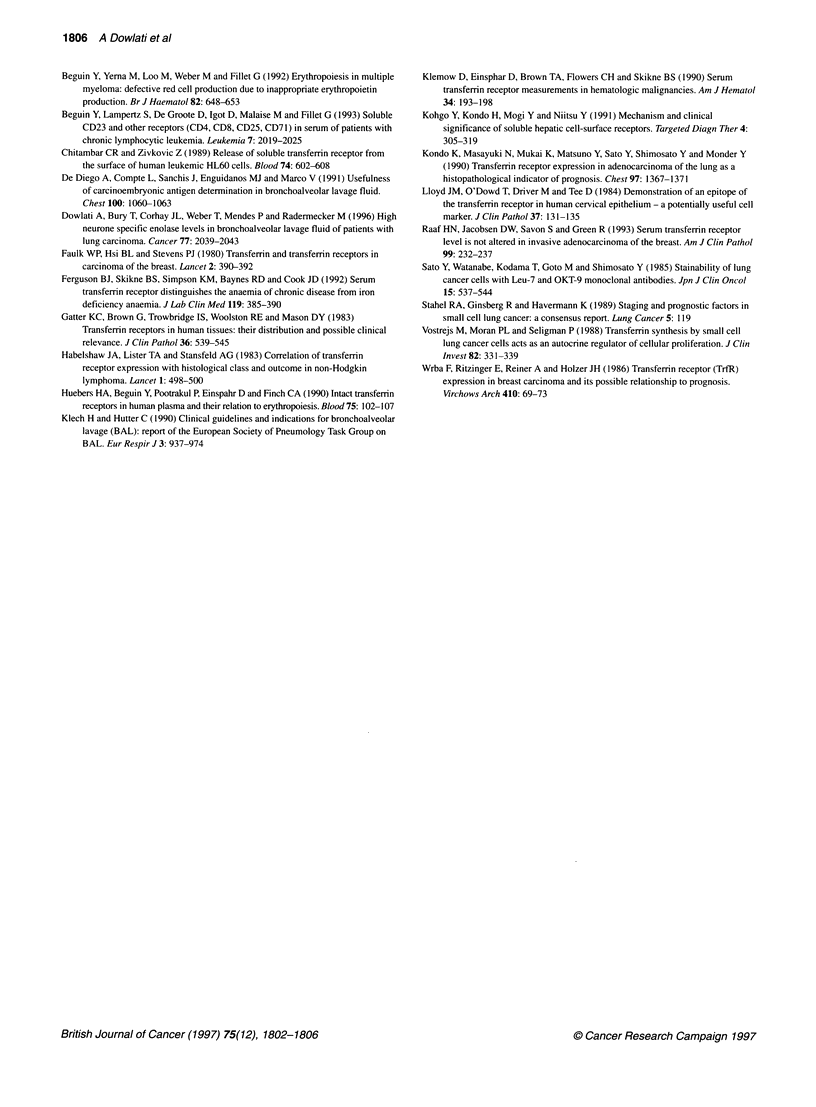# Soluble and cell-associated transferrin receptor in lung cancer.

**DOI:** 10.1038/bjc.1997.307

**Published:** 1997

**Authors:** A. Dowlati, M. Loo, T. Bury, G. Fillet, Y. Beguin

**Affiliations:** Department of Internal Medicine, University of LiÃ¨ge, School of Medicine, Belgium.

## Abstract

The expression of transferrin receptor (TfR) has been identified in many malignant tumours. In lung cancer, lymphoma and breast cancer, it has been shown that the expression of TfR correlates with tumour differentiation, probably implying some prognostic value. A soluble form of TfR (sTfR) in human serum has been shown to be proportional to the number of cellular TfRs. Based on these data we examined the utility of measuring sTfR in the serum and bronchoalveolar lavage (BAL) fluid of patients with lung cancer (n = 32) and patients with chronic obstructive pulmonary disease (n = 22). BAL fluid was centrifuged to separate the supernatant from the cellular component. Cells were lysed in a detergent and cell-associated TfR was measured by enzyme-linked immunosorbent assay (ELISA) and expressed as ng 10(-6) cells in this cellular component. There was no difference in serum sTfR between the cancer and chronic obstructive pulmonary disease (COPD) groups. A higher level of cell-associated TfR was found in BAL of non-small-cell lung cancer patients than in COPD patients (P = 0.01). The calculated number of TfR molecules per cell in BAL correlated positively with the percentage of macrophages in BAL (P < 0.0001), suggesting that cell-associated TfR in BAL originates primarily from macrophages in this fluid. No correlation existed between BAL cell-associated TfR and tumour size, nodal status, the presence of metastases and serum sTfR. BAL cell-associated TfR was negatively correlated with BAL supernatant neuron-specific enolase (NSE) (P = 0.01). A combination of BAL supernatant NSE and cell-associated TfR detected lung cancer with a sensitivity of 91%, a specificity of 59% and positive and negative predictive values of 81% and 71% respectively. In conclusion, BAL cell-associated TfR may help in the differential diagnosis of lung cancer vs pneumonia.


British Journal of Cancer (1997) 75(12), 1802–1806
© 1997 Cancer Research Campaign

### Soluble and cell-associated transferrin receptor in lung cancer

**A Dowlati[1]*, M Loo[1], T Bury[2], G Fillet[1] and Yves Beguin[1]**

Department of Internal Medicine, Divisions of [1]Haematology/Oncology and [2]Respiratory Medicine, University of Liège, School of Medicine, Liège, Belgium

**Summary** The expression of transferrin receptor (TfR) has been identified in many malignant tumours. In lung cancer, lymphoma and breast cancer, it has been shown that the expression of TfR correlates with tumour differentiation, probably implying some prognostic value. A soluble form of TfR (sTfR) in human serum has been shown to be proportional to the number of cellular TfRs. Based on these data we examined the utility of measuring sTfR in the serum and bronchoalveolar lavage (BAL) fluid of patients with lung cancer ($n$ = 32) and patients with chronic obstructive pulmonary disease ($n$ = 22). BAL fluid was centrifuged to separate the supernatant from the cellular component. Cells were lysed in a detergent and cell-associated TfR was measured by enzyme-linked immunosorbent assay (ELISA) and expressed as ng $10^{-6}$ cells in this cellular component. There was no difference in serum sTfR between the cancer and chronic obstructive pulmonary disease (COPD) groups. A higher level of cell-associated TfR was found in BAL of non-small-cell lung cancer patients than in COPD patients ($P$ = 0.01). The calculated number of TfR molecules per cell in BAL correlated positively with the percentage of macrophages in BAL ($P$ < 0.0001), suggesting that cell-associated TfR in BAL originates primarily from macrophages in this fluid. No correlation existed between BAL cell-associated TfR and tumour size, nodal status, the presence of metastases and serum sTfR. BAL cell-associated TfR was negatively correlated with BAL supernatant neuron-specific enolase (NSE) ($P$ = 0.01). A combination of BAL supernatant NSE and cell-associated TfR detected lung cancer with a sensitivity of 91%, a specificity of 59% and positive and negative predictive values of 81% and 71% respectively. In conclusion, BAL cell-associated TfR may help in the differential diagnosis of lung cancer vs pneumonia.

**Keywords:** lung neoplasm; transferrin receptor; bronchoalveolar lavage

Iron transport in the plasma is carried out by transferrin, which donates iron to cells through its interaction with a specific membrane receptor, the transferrin receptor (TfR). Immunohistochemical staining for TfR has been used most extensively for measuring the proliferation rate of cells and is thought to be of some prognostic value in several types of malignant tumours (Faulk et al, 1980; Habelshaw et al, 1983; Wrba et al, 1986). It has also been demonstrated that TfR expression, detected by the monoclonal antibody OKT9, is correlated in pulmonary adenocarcinoma with the degree of histological differentiation, the degree of nuclear atypia and mitotic index and is thus important in prognosis of these malignant tumours (Kondo et al, 1990). A soluble form of TfR (sTfR) in human serum has been shown to be proportional to the number of cellular TfRs (Chitambar et al, 1989). Furthermore, it has recently been demonstrated that bronchoalveolar lavage (BAL) fluid is a suitable place to measure certain markers for lung cancer (De Diego et al, 1991; Dowlati et al, 1996).

Based on these preliminary data we investigated the utility of measuring sTfR in the serum and bronchoalveolar lavage fluid of patients with lung cancer. To appreciate the diagnostic value of TfR measurements we compared them with values obtained in a group of chronic obstructive pulmonary disease (COPD) patients with similar smoking habits.

*Received 31 May 1996*
*Revised 18 November 1996*
*Accepted 28 November 1996*

*Correspondence to:* Y Beguin, Division of Haematology/Oncology, CHU du Sart-Tilman, B-35, 4000 Liège, Belgium

### MATERIALS AND METHODS

### Patients

A total of 54 patients were enrolled in this study. The first group consisted of 32 patients with lung cancer, of which 23 had non-small-cell lung cancer (NSCLC) and nine had small-cell lung cancer (SCLC). All patients were newly diagnosed and none had received any kind of treatment for their cancer nor had received blood transfusions. Diagnosis of lung cancer was performed in 28 of these patients by bronchial biopsy or transbronchial forceps biopsy and in four by thoracotomy. Mean age was 66 (range 49–76) with a male to female ratio of 9:1. Thirty of the 32 patients in this group were heavy smokers. The mean smoking pack–year value among smokers was 25. The second group were patients ($n$ = 22) with chronic obstructive pulmonary disease (COPD), all of whom were heavy smokers with mean smoking pack–year value of 24. Fibreoptic bronchoscopy was performed in these patients for an episode of either infectious bronchitis or pneumonia. The diagnosis of infectious bronchitis and pneumonia was made by response to antibiotic therapy, the detection of causative bacteria on bronchial aspiration and no evidence of cancer at the 1.6-year follow-up. The mean age in this group was 62 (range 41–77) with a male to female ratio of 6:1. No difference existed in the age of the two groups or in the pack–year smoking value.

*Present address: Division of Haematology/Oncology, Case Western Reserve University, Cleveland, OH, USA.

### Fibreoptic bronchoscopy, BAL and serum collection

BAL was performed according to the European BAL Task Group norms (Klech and Hutter, 1990). The tip of the fibreoptic bronchoscope was wedged into the affected bronchus or to the one closest to the lesion in the tumour group and into the lingula or middle lobe in the group with COPD. Subsequently, 150 ml of 0.9% sterile saline serum was instilled in three aliquots of 50 ml. The fluid of each instillation was recovered by gentle suction. The total aspirated volume was transferred to the laboratory where the fluid was centrifuged at 500 ***g*** for 10 min to separate the cellular component from the supernatant. The number of cells per ml of the cellular component was then determined. Differential cell counts were performed on this cellular component. The cellular component of BAL fluid was homogenized with 1 ml of a detergent solution [phosphate-buffered saline (PBS), 2% polyoxyethylene 9 lauryl ether and protease inhibitors] for 30 s at 15 000 r.p.m. and 15 s at 24 000 r.p.m. in order to rupture the cellular membrane and release cell-associated TfR into the solution. This solution was then centrifuged at 20 000 r.p.m. for 20 min at 4°C to eliminate cell ghosts. The solubilized cell-associated TfR in this solution was measured by the enzyme linked immunosorbent assay (ELISA) method described below and expressed as ng $10^{-6}$ cells. The sTfR was also measured in the supernatant and expressed as ng per ml. Blood was drawn the same day as the BAL and serum sTfR was measured in the serum according to the ELISA method.

### Soluble TfR assay

An ELISA (Huebers et al, 1990) with minor modifications was used to measure serum and BAL fluid sTfR levels. Each sample was run in triplicate. The between-assay variability (coefficient of variation) was 7.2% when the same control sample was measured in each plate. Serum sTfR levels were available in 26 patients in the cancer group and 16 patients in the COPD group.

### Staging

Tumour staging for NSCLC was carried out using conventional imaging techniques [chest and abdominal computerized tomography (CT) scans, total body bone scintigraphy and cerebral CT scans if clinically relevant]. Assignment to T, N and M was made using the Union Internationale contre le Cancer (UICC) TNM staging system. The results of TNM staging were then classified into overall stages of I–IV according to the American Joint Committee on Cancer staging criteria (American Joint Committee on Cancer. *Lung*, 1992). The nine cases of SCLC were categorized as being either localized or extensive disease (Stahel et al, 1989). The results of clinical staging in NSCLC were as follows: T1 = 3, T2 = 4, T3 = 10, T4 = 6, N0 = 7, N1 = 4, N2 = 5, N3 = 7, M1 = 5, stage I = 1, stage II = 1, stage III = 16, stage IV = 5. In SCLC there were four cases of limited and five cases of extensive disease.

### Neuron-specific enolase (NSE) measurements

NSE measurements on the supernatant of BAL fluid were performed using a commercial kit (Cis Bio-International, Gif-sur-Yvette, France) according to the manufacturer's instructions. Lactate dehydrogenase (LDH) levels were determined by the photometric method using a commercial kit (Merck, Darmstadt, Germany). Results of NSE in BAL fluid were expressed as nanograms (ng) per 100 international units (IU) of LDH. The values of BAL fluid NSE expressed as ng $100^{-1}$ IU LDH were calculated as individual ratio values; that is the absolute value of NSE in BAL was multiplied by 100 and then divided by the absolute value of LDH in the BAL of each subject. This was done in order to compensate the diluting effect of BAL.

### Statistics

All data are expressed as means ± standard deviation. Values of serum sTfR were transformed to their corresponding logarithmic values in order to achieve normality in distribution. Student's *t*-test was used to assess the significance of differences between the groups. A probability of 0.05 was considered significant. Linear regression and Spearman's correlation coefficient were used to assess association between some of the parameters. ANOVA was used to detect differences in sTfR measurements between different T, N and M groups. Sensitivity, specificity and predictive values of sTfR and NSE measurements were determined using 2 × 2 contingency tables. The log-rank test was used to compare survival rates between the appropriate groups.

### RESULTS

Levels of TfR solubilized from the cellular component of BAL fluid (expressed as ng $10^{-6}$ cells) are shown in Figure 1. There was a significant difference in BAL fluid cell-associated TfR levels between NSCLC (612 ± 337) and COPD (353 ± 337, $P = 0.01$). There was also a significant difference between NSCLC and SCLC (330 ± 369, $P = 0.04$). There was no difference between SCLC and COPD ($P = 0.9$). The median percentage of macrophages in the cellular component of BAL fluid for NSCLC was 75 (range 25–96) for SCLC 54 (range 4–97) and for COPD 72 (range 2–100). The difference in the percentage of macrophages between the three groups was statistically non-significant ($P = 0.18$). In order to elucidate the origin of TfR solubilized from the cellular component of BAL fluid, correlation was sought between the percentage of macrophages in BAL and the calculated number of molecules of TfR per cell in this fluid. In the cancer group a highly significant correlation existed between the percentage of

**Figure 1** Levels of cell-associated TfR in BAL fluid expressed as ng $10^{-6}$ cells. A significantly higher level is found in NSCLC than in COPD ($P = 0.01$) and in SCLC ($P = 0.04$)

© Cancer Research Campaign 1997

Figure 2 Correlation between the number of calculated molecules of TfR per cell in the cellular component of BAL and the percentage of macrophages in this fluid. This correlation is highly significant ($r = 0.73$, $P < 0.0001$)

Figure 3 Correlation between the cell-associated TfR levels in BAL and NSE levels in the supernatant of BAL. The correlation is negative and statistically significant ($r = -0.423$, $P = 0.01$)

macrophages and the number of molecules of TfR per cell in the BAL cellular component ($r = 0.73$, $P \leq 0.001$, Figure 2). Indeed very little cell-associated TfR was detected when the percentage of macrophages was below 50%; accordingly, when samples with less than 50% were excluded from the analysis the correlation between the number of molecules of TfR per cell and the percentage of macrophages became non-significant ($r = 0.29$, $P = 0.17$), suggesting that a critical number of macrophages must be present in the cellular component of BAL fluid in order to have a significant level of cell-associated TfR. This correlation was also significant for the COPD group ($r = 0.49$, $P = 0.02$). When cell-associated TfR in BAL fluid was expressed as ng $10^{-6}$ macrophages the difference between NSCLC (881 ± 357) and COPD (564 ± 414) remained significant ($P = 0.01$); however, the difference between NSCLC and SCLC (655 ± 356) became non-significant ($P = 0.10$). No correlation was found between the absolute numbers of macrophages in BAL per ml (222 640 ± 436 000) and BAL cell-associated TfR ($P = 0.7$). No correlation existed between BAL cell-associated TfR and tumour size ($P = 0.3$), nodal status ($P = 0.7$) and the presence of metastases ($P = 0.2$).

BAL supernatant sTfR was also measured. Because the limit of detection of the current ELISA method for sTfR is 2 ng ml$^{-1}$, 8 of 30 cancer patients (levels were available in 30 of 32 cancer patients) had non-dosable levels of sTfR in the BAL supernatant and 22 had values ≥ 10 ng ml$^{-1}$ (30.3 ± 45.6). The correlation between sTfR in the supernatant of BAL and cell-associated TfR in BAL did not reach statistical significance ($P = 0.07$).

The serum levels of sTfR (μg l$^{-1}$) were as follows: NSCLC (2932 ± 1950), SCLC (2695 ± 687) and COPD (3213 ± 855). Based on previous studies the value of serum sTfR in normal individuals is 5000 ± 1100 ng ml$^{-1}$ (Beguin et al, 1992*a*). No difference could be found between the three groups in our study ($P = 0.9$). Furthermore, there was no relation between serum sTfR and BAL supernatant or cell-associated TfR. In the cancer patients, no correlation was found between serum sTfR on the one hand and serum ferritin (255 ± 155 μg l$^{-1}$, $P = 0.2$), TIBC (41.3 ± 9.5 μmol l$^{-1}$, $P = 0.5$) or serum iron (12.1 ± 7.8 μmol l$^{-1}$, $P = 0.1$) levels on the other. Only five patients had serum ferritin below 100 μg l$^{-1}$, the lowest value being 50 μg ml$^{-1}$. Based on serum ferritin levels, patients with cancer were divided into two groups: ≥ 200 or < 200 pmol l$^{-1}$. No difference was found in the serum sTfR between these two groups ($P = 0.5$).

Results of NSE in the supernatant of BAL fluid of the corresponding patients can be found in a previous article (Dowlati et al, 1996). Comparing the results of cell-associated TfR in BAL and NSE in BAL supernatant (expressed as ng per 100 IU of LDH), we found a significant negative correlation between the two parameters ($r = -0.423$, $P = 0.01$, Figure 3). Using 500 ng $10^{-6}$ cells as the cut-off point, the sensitivity and specificity of BAL cell-associated TfR for detecting NSCLC were 65% and 77% respectively. The positive and negative predictive values were 75% and 68% respectively. Using a combination of cell-associated TfR in BAL (at the cut-off point mentioned above) and a cut-off point for BAL supernatant NSE of 6.6 ng per 100 IU of LDH, the sensitivity and specificity for the diagnosis of NSCLC were 91% and 59% respectively. Negative and positive predictive values were 81% and 71% respectively. Interestingly, BAL fluid NSE had a negative correlation with the percentage of BAL fluid macrophages ($r = -0.3551$, $P = 0.03$).

Using a cut-off point of 750 ng $10^{-6}$ cells of cell-associated TfR in BAL, patients with stage III and IV lung cancer were divided into two groups. No survival difference was seen between these two groups (log-rank test, $P = 0.57$, Figure 4).

### DISCUSSION

Transferrin functions to maintain cellular proliferation by providing iron for processes that have yet to be defined. It has been demonstrated that transferrin synthesis by SCLC acts as an autocrine regulator of cellular proliferation (Vostrejs et al, 1988). Immunohistochemical staining for transferrin receptor, which is needed for internalization of transferrin and iron, has been used most extensively for measuring the proliferation rate of cells and is thought to be of some prognostic value in several types of malignant tumours. All cells except mature red blood cells express the transferrin receptor. Since Faulk et al (1980) reported that TfR was observed in breast cancer, the expression of TfR has been identified in many types of malignant tumours including malignant lymphoma (Habelshaw et al, 1983), gastric cancer (Gatter et al, 1983), uterine cancer (Lloyd et al, 1981) and lung cancer

 © Cancer Research Campaign 1997

**Figure 4** Comparison of survival in patients with stage III or IV NSCLC with cell-associated TfR levels in BAL ≥750 ng $10^{-6}$ cells or <750 ng $10^{-6}$ cells. No survival difference is seen (log-rank test, $P = 0.57$)

(Sato et al, 1985). In malignant lymphoma (Habelshaw et al, 1983) and breast cancer (Faulk et al, 1980; Wrba et al, 1986), it has been shown that the expression of TfR correlates with tumour differentiation, probably implying some prognostic significance. It has also been demonstrated that TfR expression, detected by the monoclonal antibody OKT9, is correlated in pulmonary adenocarcinoma with the degree of histological differentiation, the degree of nuclear atypia and mitotic index, and may thus be important in prognosis of the malignant tumours (Kondo et al, 1990). A soluble form of TfR has also been detected in the serum. An excellent correlation between cellular TfR and soluble TfR (sTfR) has been demonstrated both in vivo and by incubations of tumour cell lines (Chitamber et al, 1989). Based on these data, we measured serum sTfR levels as well as supernatant sTfR and cell-associated TfR in BAL fluid of patients with lung cancer and compared them with values obtained in a group of COPD patients. COPD patients were used as the control group because they have similar smoking habits to lung cancer patients.

Our study shows that cell-associated TfR levels are significantly higher in the BAL fluid of NSCLC patients than in SCLC and COPD patients. The study by Kondo et al (1990) also showed high expression of cellular TfR in adenocarcinoma of the lung. However, our study indicates that cell-associated TfR in BAL fluid probably originates mainly from macrophages found in this fluid and not directly from tumour cells. We have also demonstrated that cell-associated TfR levels in BAL fluid are negatively correlated with BAL NSE levels and that BAL NSE levels are inversely related to the percentage of macrophages in this fluid. Furthermore, our previous study suggests that BAL fluid levels of NSE are a reflection of their local tumour cell expression, which is not the case for cell-associated TfR (Dowlati et al, 1996). The correlation between BAL supernatant sTfR and cell-associated TfR levels did not reach statistical significance, probably because values in supernatant were very low and thus less precise.

BAL fluid analysis for markers of lung cancer has recently been shown to be interesting. De Diego et al (1991) showed that the combined measurements of serum CEA and BAL fluid CEA has sensitivity and specificity of 88% for lung cancer with a positive predictive value of 66% when compared with patients with pneumonia. We have previously shown that NSE in BAL fluid is a better predictor of malignancy than serum NSE and that no relation exists between serum and BAL levels of this marker (Dowlati et al, 1996). The findings in this study further confirm that levels of markers such as TfR and NSE (Dowlati et al, 1996) in BAL fluid have no relation to their corresponding serum levels. In addition, cell-associated TfR proved to have predictive value for the diagnosis of cancer and that the sensitivity of this prediction could be improved by combining it with BAL supernatant NSE levels. The combination of multiple markers in BAL may thus contribute to the diagnosis of peripheral lung cancers for which obtaining histological diagnosis is difficult.

Although tissue expression of TfR has been shown to be correlated with the degree of histological differentiation, the degree of nuclear atypia and mitotic index, it has not been shown that this is an independent prognostic factor. Accordingly, our study has failed to show a survival difference between patients with high cell-associated TfR in the cellular component of BAL fluid and patients with lower levels. This might be due to the limited number of patients in our study and we believe that a study with a larger number of patients should be conducted to clarify the prognostic significance of cell-associated TfR in BAL fluid of NSCLC patients.

In our study serum sTfR levels were 'low'. In a group of 165 normal human subjects, receptor levels in the serum averaged 5000 ± 1100 ng $ml^{-1}$ (Beguin, 1992). In the serum sTfR levels are essentially a reflection of erythropoiesis (Huebers et al, 1990). Consequently, in the presence of anaemia of chronic disease, patients with solid tumours and multiple myeloma may have normal or low levels of serum sTfR (Beguin et al, 1992; Raaf et al, 1993). However, levels of serum sTfR are increased in chronic lymphocytic leukaemia (Beguin et al, 1993) and erythroid malignancies (Huebers et al, 1990, Klemow et al, 1990), and possibly hepatocarcinoma (Kohgo et al, 1991). Our study thus shows that serum sTfR levels are not increased in lung cancer and that they do not differ from levels in COPD patients. The lower levels of serum sTfR in our series of lung cancer patients can be interpreted as a state of erythroid hypoplasia in the context of anaemia of chronic disease and can rule out iron deficiency (Ferguson et al, 1992). No correlation was seen between serum sTfR and ferritin, TIBC or serum iron. Because serum ferritin levels are frequently elevated in advanced cancer, using ferritin alone for the diagnosis of iron deficiency is not reliable. However, as very few patients had serum ferritin levels < 100 μg $l^{-1}$ and none had elevated serum sTfR levels, iron deficiency can be excluded fairly in our patients.

### ACKNOWLEDGEMENTS

Martine Loo was supported by research fellowships from 'Télévie' (National Fund for Scientific Research) and 'Fondation Frédérick' (University of Liège). Yves Beguin is a Senior Research Associate of the National Fund for Scientific Research (FNRS, Belgium). This work was supported in part by grants no. 3.4555.91 of the FRSM (Fund for Medical Scientific Research) and no. 3.4621.94 of the FNRS.

### REFERENCES

American Joint Committee on Cancer. Lung (1992) Manual for the staging of cancer, Beahrs OH, Henson DE and Hutter RVP (eds), pp. 115–122. JB Lippincott: Philadelphia

Beguin Y (1992) The soluble transferrin receptor: biological aspects and clinical usefulness as quantitative measure of erythropoiesis. *Haematologica* **77**: 1–10

Beguin Y, Yerna M, Loo M, Weber M and Fillet G (1992) Erythropoiesis in multiple myeloma: defective red cell production due to inappropriate erythropoietin production. *Br J Haematol* **82**: 648–653

Beguin Y, Lampertz S, De Groote D, Igot D, Malaise M and Fillet G (1993) Soluble CD23 and other receptors (CD4, CD8, CD25, CD71) in serum of patients with chronic lymphocytic leukemia. *Leukemia* **7**: 2019–2025

Chitambar CR and Zivkovic Z (1989) Release of soluble transferrin receptor from the surface of human leukemic HL60 cells. *Blood* **74**: 602–608

De Diego A, Compte L, Sanchis J, Enguidanos MJ and Marco V (1991) Usefulness of carcinoembryonic antigen determination in bronchoalveolar lavage fluid. *Chest* **100**: 1060–1063

Dowlati A, Bury T, Corhay JL, Weber T, Mendes P and Radermecker M (1996) High neurone specific enolase levels in bronchoalveolar lavage fluid of patients with lung carcinoma. *Cancer* **77**: 2039–2043

Faulk WP, Hsi BL and Stevens PJ (1980) Transferrin and transferrin receptors in carcinoma of the breast. *Lancet* **2**: 390–392

Ferguson BJ, Skikne BS, Simpson KM, Baynes RD and Cook JD (1992) Serum transferrin receptor distinguishes the anaemia of chronic disease from iron deficiency anaemia. *J Lab Clin Med* **119**: 385–390

Gatter KC, Brown G, Trowbridge IS, Woolston RE and Mason DY (1983) Transferrin receptors in human tissues: their distribution and possible clinical relevance. *J Clin Pathol* **36**: 539–545

Habelshaw JA, Lister TA and Stansfeld AG (1983) Correlation of transferrin receptor expression with histological class and outcome in non-Hodgkin lymphoma. *Lancet* **1**: 498–500

Huebers HA, Beguin Y, Pootrakul P, Einspahr D and Finch CA (1990) Intact transferrin receptors in human plasma and their relation to erythropoiesis. *Blood* **75**: 102–107

Klech H and Hutter C (1990) Clinical guidelines and indications for bronchoalveolar lavage (BAL): report of the European Society of Pneumology Task Group on BAL. *Eur Respir J* **3**: 937–974

Klemow D, Einsphar D, Brown TA, Flowers CH and Skikne BS (1990) Serum transferrin receptor measurements in hematologic malignancies. *Am J Hematol* **34**: 193–198

Kohgo Y, Kondo H, Mogi Y and Niitsu Y (1991) Mechanism and clinical significance of soluble hepatic cell-surface receptors. *Targeted Diagn Ther* **4**: 305–319

Kondo K, Masayuki N, Mukai K, Matsuno Y, Sato Y, Shimosato Y and Monder Y (1990) Transferrin receptor expression in adenocarcinoma of the lung as a histopathological indicator of prognosis. *Chest* **97**: 1367–1371

Lloyd JM, O'Dowd T, Driver M and Tee D (1984) Demonstration of an epitope of the transferrin receptor in human cervical epithelium – a potentially useful cell marker. *J Clin Pathol* **37**: 131–135

Raaf HN, Jacobsen DW, Savon S and Green R (1993) Serum transferrin receptor level is not altered in invasive adenocarcinoma of the breast. *Am J Clin Pathol* **99**: 232–237

Sato Y, Watanabe, Kodama T, Goto M and Shimosato Y (1985) Stainability of lung cancer cells with Leu-7 and OKT-9 monoclonal antibodies. *Jpn J Clin Oncol* **15**: 537–544

Stahel RA, Ginsberg R and Havermann K (1989) Staging and prognostic factors in small cell lung cancer: a consensus report. *Lung Cancer* **5**: 119

Vostrejs M, Moran PL and Seligman P (1988) Transferrin synthesis by small cell lung cancer cells acts as an autocrine regulator of cellular proliferation. *J Clin Invest* **82**: 331–339

Wrba F, Ritzinger E, Reiner A and Holzer JH (1986) Transferrin receptor (TrfR) expression in breast carcinoma and its possible relationship to prognosis. *Virchows Arch* **410**: 69–73

© Cancer Research Campaign 1997